# Quantitative CT perfusion imaging in patients with pancreatic cancer: a systematic review

**DOI:** 10.1007/s00261-021-03190-w

**Published:** 2021-07-05

**Authors:** T. H. Perik, E. A. J. van Genugten, E. H. J. G. Aarntzen, E. J. Smit, H. J. Huisman, J. J. Hermans

**Affiliations:** Department of Medical Imaging, Radboud University Medical Center, P.O. Box 9101, 6500 HB Nijmegen, The Netherlands

**Keywords:** CT perfusion, Adenocarcinoma, Pancreas, Quantitative imaging

## Abstract

Pancreatic ductal adenocarcinoma (PDAC) is the third leading cause of cancer-related death with a 5-year survival rate of 10%. Quantitative CT perfusion (CTP) can provide additional diagnostic information compared to the limited accuracy of the current standard, contrast-enhanced CT (CECT). This systematic review evaluates CTP for diagnosis, grading, and treatment assessment of PDAC. The secondary goal is to provide an overview of scan protocols and perfusion models used for CTP in PDAC. The search strategy combined synonyms for ‘CTP’ and ‘PDAC.’ Pubmed, Embase, and Web of Science were systematically searched from January 2000 to December 2020 for studies using CTP to evaluate PDAC. The risk of bias was assessed using QUADAS-2. 607 abstracts were screened, of which 29 were selected for full-text eligibility. 21 studies were included in the final analysis with a total of 760 patients. All studies comparing PDAC with non-tumorous parenchyma found significant CTP-based differences in blood flow (BF) and blood volume (BV). Two studies found significant differences between pathological grades. Two other studies showed that BF could predict neoadjuvant treatment response. A wide variety in kinetic models and acquisition protocol was found among included studies. Quantitative CTP shows a potential benefit in PDAC diagnosis and can serve as a tool for pathological grading and treatment assessment; however, clinical evidence is still limited. To improve clinical use, standardized acquisition and reconstruction parameters are necessary for interchangeability of the perfusion parameters.

**Graphic abstract:**

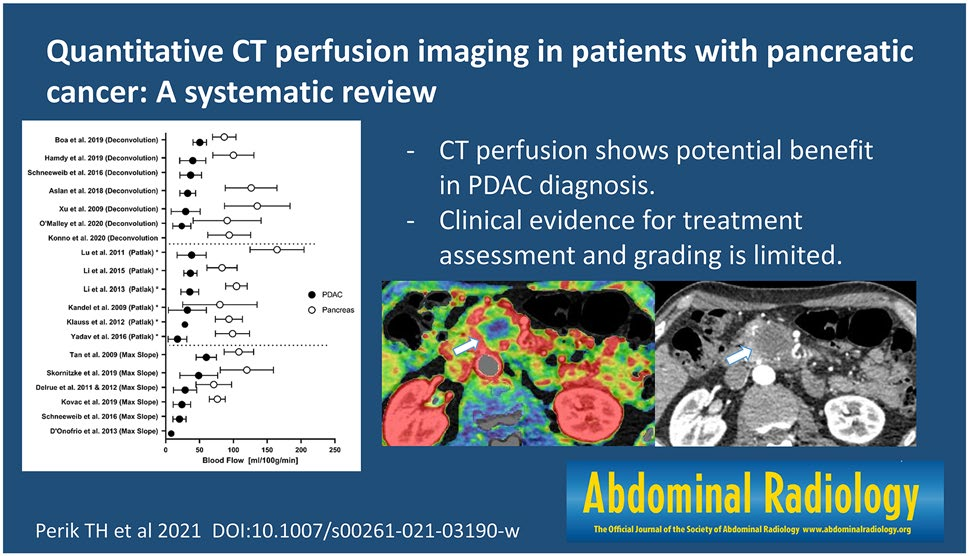

**Supplementary Information:**

The online version contains supplementary material available at 10.1007/s00261-021-03190-w.

## Introduction

Pancreatic ductal adenocarcinoma (PDAC) is the third leading cause of cancer-related death in the USA [1]. The incidence increases by an estimated 0.5% per year, while prognosis remains poor, with a 5-year survival rate of 10% [1]. Availability and advancement of imaging technology and new treatment options have not improved 5-year survival in the last 40 years, mainly due to late presentation [2]. As 40% presents with locally advanced disease and 40–45% with metastatic disease, the majority of patients face incurable disease [3].

Biphasic or triphasic contrast-enhanced CT (CECT), with at least both an arterial and portal-venous phase, is the current standard for diagnosis, assessment of resectability, and monitoring of therapy of PDAC [4]. However, several problems are currently encountered using CECT. First, tumor characterization and delineation, essential for staging and pre-operative planning, are not always possible. Accurate diagnosis based on CECT is difficult as tumors may appear isoattenuating to the surrounding parenchyma in 15–20% of cases [5, 6]. In addition, inflammatory masses of acute and chronic pancreatitis can mimic PDAC on CECT, risking misdiagnosis [7]. Second, histopathological analysis is the gold standard, and tumor grading is an important parameter of survival [8]. However, biopsies do not always yield sufficient material for pathological grade analysis. With fine-needle biopsy (FNB), accurate histology is retrieved in 77% of all procedures, at the risk of complications [9]. Furthermore, as PDAC is a heterogeneous tumor, biopsy could lead to a sampling error [10]. A noninvasive, reliable imaging biomarker would be highly desirable to accurately assess the histological grading of the complete tumor, which could aid in selecting patients for the appropriate treatment. Third, with the current imaging techniques, RECIST-based treatment assessment is insufficient. Neoadjuvant chemotherapy is increasingly applied for potential resectable PDAC [11]. However, RECIST-based restaging remains troublesome because changes in tumor size and vessel encasement using CECT prove to be inadequate for reliable response assessment after neoadjuvant treatment [12]. Moreover, the correlation between RECIST and histopathological grading of tumor response is poor [13]. Thus, CECT response criteria could underestimate the treatment response, showing the need for a more reliable predictor to increase the amount of margin-negative resections (i.e., R0).

CT perfusion (CTP), in other words dynamic contrast-enhanced CT, is a novel modality that could improve the diagnostic workup of PDAC by combining functional information and spatial detail [14]. CTP is an imaging technique where dynamic acquisition after injection of a contrast agent enables quantification of tissue vascularization [15]. Using a kinetic model, parameters can be calculated, which reflect intratumoral differences in perfusion and vascular permeability. Therefore, CTP bears potential as a biomarker in oncology for tumor angiogenesis, enabling prediction of tumor grading and assessment of treatment response [16–19]. Over the last years, CTP is increasingly utilized as a functional imaging biomarker, and several articles reported additional benefits and advances of CTP in pancreatic cancer.

This systematic review aims to evaluate the literature on CTP in pancreatic cancer for diagnosis, grading, and treatment assessment. The secondary goal is to provide an overview of scan protocols and perfusion models used for CTP in the pancreas.

## Materials and methods

### Search strategy

This systematic review was carried out in accordance with the Preferred Reporting Items for Systematic Reviews and Meta-Analysis 2015 (Prisma-P 2015) [20]. The protocol for this systematic review is registered in the PROSPERO database [Registration number: CRD42021213438] [21]. The search strategy combined synonyms for ‘CTP,’ ‘Dynamic contrast-enhanced CT,’ ‘Pancreatic cancer,’ and ‘Pancreatic adenocarcinoma’; the complete search is accessible in the Supplementary materials. This systematic search was performed in the following libraries: PubMed, EMBASE, and Web of Science; search terms were tailored to each database. The timeframe for published articles was 1 January 2000–31 December 2020.

### Eligibility criteria

Studies were considered eligible when CTP was used during diagnosis or treatment assessment of primary PDAC. The scan protocol must be described clearly and consists of dynamic acquisitions resulting in calculated perfusion parameters, such as blood flow (BF), blood volume (BV), and permeability surface area product (PS).

### Study selection

Retrieved articles were imported into EndNote and duplicates removed. The article titles and abstracts resulting from the search were screened independently by two reviewers (T.P, E.G.). Studies meeting the inclusion criteria were selected for full-text screening and reviewed. In the subsequent full-text screening stage, any disagreements were resolved by consensus, and if consensus was not reached, a third reviewer (J.H.) was consulted.

### Data extraction/synthesis

Relevant study characteristics and scan parameters were extracted by one reviewer and checked by the second reviewer. Scan parameters included type of detector, number of acquisitions, contrast injection, and type of kinetic model. Comparison of studies was performed using perfusion values such as BF, BV, and PS. To compare different studies, perfusion parameters were converted to mL/100 g/min when reported in mL/100 mL/min using a tissue density of 1.05 gr/mL [15]. Due to heterogeneity in the data, only descriptive statistic were applied. Studies were categorized based on the goal of the study and the type of kinetic model. Graphs were created using GraphPad Prism 9.

### Quality assessment

The risk of bias and applicability of each study were assessed using the Quality Assessment of Diagnostic Accuracy Studies (QUADAS-2) tool. The risk of bias and applicability concerns is defined as the risk to deviate from the QUADAS-2 guidelines described in four domains: patient selection, index test, reference standard, and flow and timing. QUADAS signal questions regarding index test were adjusted to classify description of perfusion scan protocol, kinetic model software, and ROI measurement. Consensus about the assessment was reached between the same two reviewers who selected studies for inclusion.

## Results

### Study selection

With the described search strategy, 881 articles were identified, which were reduced to 607 articles after removing duplicates. 607 articles were screened by title and abstracts, resulting in 29 articles eligible for full-text screening. Eight full-text articles were excluded as they did not meet the final inclusion criteria. Finally, 21 articles were included in the qualitative analysis, with a total of 760 patients with PDAC. All included studies with scan parameters and kinetic models can be found in Table 1, and study characteristics can be found in Table 2.

**Table 1:** Overview of included studies with reported scan parameters and kinetic model

Author, publication year, citation	Contrast injection (I/mg)	Multislice detector (z-coverage)	kVp	mAs	Number of acquisitions (scan time)	Scan delay	Kinetic model	Software (vendor)	ROI (size)	Breathing
Aslan et al. 2018, [22]	50 mL with 5 mL/s	64 slice (110 mm)	100	100	242 slices (126 s)	0 s	Deconvolution	Syngo MMWP (Siemens)	Circular ROI (15mm^2^)	Shallow breathing
Bao et al. 2019, [30]	50 mL with 5 mL/s	64 slice (84 mm)	80	200	17 acq (49 s)	7 s	Deconvolution	Syngo MMWP (Siemens)	3 circular ROIs (10 mm)	Abdominal belt
Delrue et al. 2011, [36]	50 mL with 5 mL/s (320)	128 slice (148 mm)	100	145	18 acq (51 s)	NA	Max slope	Syngo MMWP (Siemens)	Manual ROI (20–25 mm^2^) (3 ROI/lesion)	Oxygen hyperventilation (breath-hold 51 s)
Delrue et al. 2012, [37]	60 mL with 8 mL/s	128 slice (148 mm)	100	145	18 acq (51 s)	NA	Max slope	AW CTP 4D (GE)	Manual ROI	Oxygen hyperventilation (breath-hold 51 s)
D'Onofrio et al. 2013, [40]	50 mL with 5 mL/s	64 slice (100 mm)	120	150	12 acq (120 s)	12 s	Max slope + Patlak	AW CTP 4D (GE)	Manual ROI (max diameter tumor) + 6 small ROI	Abdominal belt
Hamdy et al. 2019, [35]	50 mL with 5 mL/s (370)	320 slice (224 mm)	120	Automated	40 acq (60 s)	2 s	Deconvolution	Syngo MMWP (Siemens)	Manual ROI (10mm^2^)(3 ROI/lesion)	Free breathing
Kandel et al. 2009, [27]	60 mL with 8 mL/s	320 slice (160 mm)	100	45	19 acq (80 s)	Bolus track	Max slope	In-house developed	Volume ROI largest diameter tumor	Breath-hold 40 s
Klauss et al. 2012, [28]	80 mL with 5 mL/s (370)	64 slice (16.8 mm)	80	270	34 acq (51 s)	5 s	Patlak	Syngo MMWP (Siemens)	Polygonal ROI	Shallow breathing
Konno et al. 2020, [44]	700 mg I/kg at 3.5–4 mL/s (370 or 350)	320 slice (160 mm)	100	35	19 acq (155 s)^a^	6 s	Deconvolution	Ziostation2 (Ziosoft)	Volume ROI (diam > 10 mm)	Abdominal belt + shallow breathing
Kovac et al. 2019, [24]	50 mL with 7 mL/s (370)	64 slice (32 mm)	100	50	25 acq (50 s)	5 s	Max slope + Patlak	In-house developed	Circular ROI in 4 slices(largest diameter)	Shallow breathing
Li et al. 2013, [32]	50 mL with 5.5 mL/s (350)	128 slice (60 mm)	< 70 kg: 70 > 70 kg: 80	< 70 kg: 120 > 70 kg: 100	24 acq (38 s)	8 s	Patlak	Syngo MMWP (Siemens)	Manual ROI (15 mm diameter)	Abdominal belt + shallow breathing
Li et al. 2015, [26]	50 mL with 5.5 mL/s (350)	320 slice (70 mm)	80	100	23 acq:(38 s)	8 s	Patlak	Syngo MMWP (Siemens)	Manual ROI (15 mm diameter)	Abdominal belt + shallow breathing
Lu et al. 2011, [31]	50 mL with 5 mL/s (300)	64 slice (28 mm)	80	100	50 acq (50 s)	–	Max slope + Patlak	Syngo MMWP (Siemens)	ROI (50–100 pixels)	Abdominal belt Shallow breathing
Nishikawa et al. 2014, [42]	40 mL with 4 mL/s (350)	64 slice	80	40	108 acq (54 s)	3 s	Max slope	Ziostation2 (Ziosoft)	Manual ROI in peritumoral tissue	Breath-hold
O’Malley et al. 2020, [25]	Weight-based at 5 mL/s (350)	256 slice (160 mm)	100	140	15 acq (38 s)^a^	5 s	Deconvolution	AW CTP 4D (GE)	Manual 3 ROIs (largest diameter tumor, center, rim)	Shallow breathing
Park et al. 2009, [41]	50 mL with 5 mL/s (300)	64 slice	80	100	30 acq (30 s)	5 s	Patlak	Syngo MMWP (Siemens)	Freehand ROI	Breath-hold
Skornitzke et al. 2019, [29]	80 mL with 5 mL/s (370)	64 slice (19.2 mm)	80	Automated	34 acq (51 s)	13 s	Max slope	Syngo MMWP (Siemens)	Polygonal ROI	Shallow breathing
Schneeweiβ et al. 2016, [39]	50 mL with 5 mL/s	128 slice (69 mm)	80	100/120	26 (40 s)	7	Max slope + Patlak & Deconvolution	Syngo MMWP (Siemens)	Volume ROI as large as possible	Shallow breathing
Tan et al. 2009, [34]	40 mL with 5 mL/s	64 slice (160 mm)	100	50	12 acq (36 s)	0	Max slope	In-house developed	ROI 1–3 mm^2^	NA
Xu et al. 2009, [23]	50 mL with 5 mL/s	64 slice (72 mm)	80		50 acq in 50 s	–	Deconvolution	Syngo MMWP (Siemens)	3 ROI (15 mm) (tumor, tumor rim and pancreatic tissue)	Breath-hold
Yadav et al. 2016, [33]	40 mL with 5 mL/s	256 slice (70-100 mm)	100	100	20 acq (95 s)	–	Patlak	Syngo MMWP (Siemens)	Single ROI center of lesion	Free breathing

^a^ Interleaved scan protocol combining CTP with a diagnostic CECT using one contrast bolus. Number of acquisitions in the perfusion protocol.

**Table 2:** Study characteristics

Author, publication year, citation	Patients with PDAC (*n* =)	Aim	Conclusion	Category	Radiation dose (mSv)	Gold standard
Aslan et al. 2018, [22]	61	Differentiate PDAC from pancreatitis in isoattenauting tumors using CTP	BV, BF, and PS are significantly lower in PDAC compared to pancreatic parenchyma. Showing CTP is useful for diagnosis	Diagnosis	6.3 ± 2.1	Pathology
Bao et al. 2019, [30]	30	Evaluate the correlation of dual-energy CT iodine maps with CTP in patients suspected of PDAC	Iodine maps are related to BF and BV obtained in CTP. Diagnostic sensitivity is lower in iodine maps compared to CTP	Diagnosis, scan technique	8.6	Pathology
Delrue et al. 2011, [36]	32	Assess CTP characteristics in patients with PDAC compared to healthy pancreatic tissue in 128-slice CT	In PDAC, BF en BV values are significantly lower than in healthy pancreatic parenchyma	Diagnosis	NA	Pathology
Delrue et al. 2012, [37]	19	Evaluate whether CTP can distinguish general pathologies of the pancreas	CTP is able to distinguish different pancreatic pathologies based on BF and BV. Showing potential for CTP in diagnosis	Diagnosis	NA	Pathology
D'Onofrio et al. 2013, [40]	32	Describe CTP parameters of locally advanced PDAC and evaluate correlation with tumor grading	Significant difference is found between high-grade and low-grade tumors for BV. CTP can predict tumor grade	Grading	NA	Pathology (grading based on differentiation)
Hamdy et al. 2019, [35]	21	Investigate the use of CTP to predict the response of PDAC to chemoradiotherapy	Higher baseline BF is a predictor of response. CTP may be useful to predict histopathological response to chemotherapy	Treatment response prediction	12.4 ± 10.8	Pathology after resection
Kandel et al. 2009, [27]	73	Evaluate whole-organ perfusion protocol of pancreas and analyze perfusion differences between normal pancreas and PDAC	PDAC shows significant lower perfusion compared to normal pancreas. CTP perfusion is feasible and shows potential as diagnostic tool	Diagnosis	10.1	Pathology
Klauss et al. 2012, [28]	25	Evaluate CTP for PDAC using the Patlak model to assess perfusion	CTP using Patlak analysis is feasible. BF, BV, and PS can be helpful to delineate PDAC	Diagnosis, scan technique	6.3	Pathology
Konno et al. 2020, [44]	17	Evaluate a volumetric CTP interleaved into pancreatic multiphasic CECT in the clinical setting	An interleaved protocol for pancreatic CTP provides high-quality imaging while requiring lower radiation dose than conventional methods	Scan technique	5.1 ± 0.3	CECT (Quality comparison)
Kovac et al. 2019, [24]	44	To evaluate CTP and DWI quantitative parameters of PDAC and to assess correlation with clinicopathological features	BV and BF are significantly lower in high-grade tumors compared to low-grade tumors. CTP could improve assessment of PDAC with possibility of grading	Grading	NA	Pathology
Li et al. 2013, [32]	46	Explore the feasibility of low-dose whole-organ CTP of pancreas in clinical application	Dose reduction in CTP is feasible. BF and BV are significantly different between PDAC and normal pancreas	Diagnosis	< 70 kg: 3.6 > 70 kg: 4.9	Pathology
Li et al. 2015, [26]	20	Investigate the value of low-dose CTP integrated with dual-energy CT in diagnosing PDAC	BF and BV are significantly different between PDAC and pancreatic parenchyma. Low-dose CTP can provide functional information	Diagnosis, scan technique	4.8–8.4	Pathology
Lu et al. 2011, [31]	64	Investigate 64-slice CTP in patients with PDAC and mass-forming chronic pancreatitis	CTP can help to distinguish PDAC from mass-forming chronic pancreatitis. BF and BV are lower in PDAC compared to pancreatitis	Diagnosis	4.8–8.4	Pathology
Nishikawa et al. 2014, [42]	30	Investigate the relationship between prognosis and perfusion in tissue surrounding PDAC using CTP	Patient prognosis may be related to perfusion in pancreatic tissue adjacent to PDAC	Treatment response prediction	NA	Survival days
O'Malley et al. 2020, [25]	57	To evaluate the feasibility of CTP combined with routine multiphase CECT in PDAC (interleaved protocol)	Combining CTP with CECT using a single-contrast injection is feasible. Perfusion parameters are in line with literature	Scan technique	10.2	Comparison with literature reference values
Park et al. 2009, [41]	17	Determine whether CTP parameters can be used to predict response to chemoradiotherapy	PDAC with higher pre-treatment permeability responds better to chemoradiotherapy	Treatment response prediction	NA	RECIST criteria
Skornitzke et al. 2019, [29]	19	Calculate CTP parameters based on quantitative iodine maps using dual-energy CT	CTP parameters can be calculated using dual-energy iodine images. Measurement accuracy is not improved compared to regular CTP	Scan Technique	8.01	Pathology
Schneeweiβ et al. 2016, [39]	48	Evaluate interchangeability of CTP parameters obtained using different kinetic models in PDAC	Significant differences in perfusion parameters are obtained using different kinetic models; however, magnitude of these parameters is correlated. Parameters do not show significant differences for histological subgroups	Kinetic models, grading	7.0(male) 7.1(female)	Pathology
Tan et al. 2009, [34]	27	Feasibility of low-dose whole-pancreas imaging utilizing 64-slice CTP	BF and tissue peak show significant differences between PDAC and pancreatic parenchyma. Reducing number of acquisitions does not significantly change perfusion parameters	Diagnosis	13.5 ± 0.5	Pathology
Xu et al. 2009, [23]	40	Explore CTP characteristics of normal pancreas and pancreatic adenocarcinoma	BV, BF, and PS in PDAC are significantly lower compared to normal pancreas. Perfusion is lower towards center of the tumor	Diagnosis	NA	Pathology
Yadav et al. 2016, [33]	42	Evaluate the use of CTP in differentiating PDAC from mass-forming chronic pancreatitis	BF, BV, and PS are significantly lower in PDAC compared to chronic pancreatitis and normal pancreas. CTP can serve as tool in diagnosis to differentiate PDAC from chronic pancreatitis	Diagnosis	NA	Pathology

**Fig. 1:**
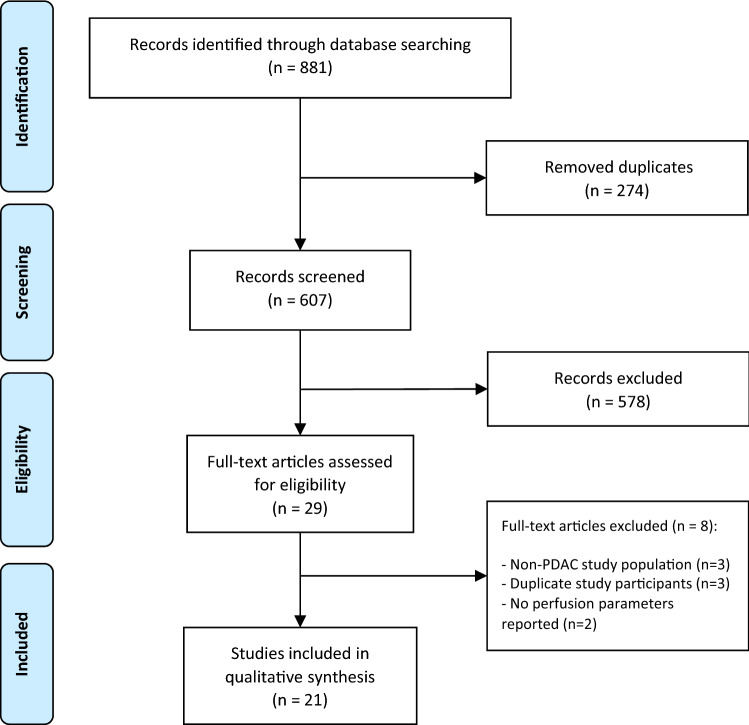
Prisma-2015 flowchart of the study selection process

The Prisma-2015 flowchart describing the selection process is visible in Fig. 1.

### Diagnosis

**Fig. 2:**
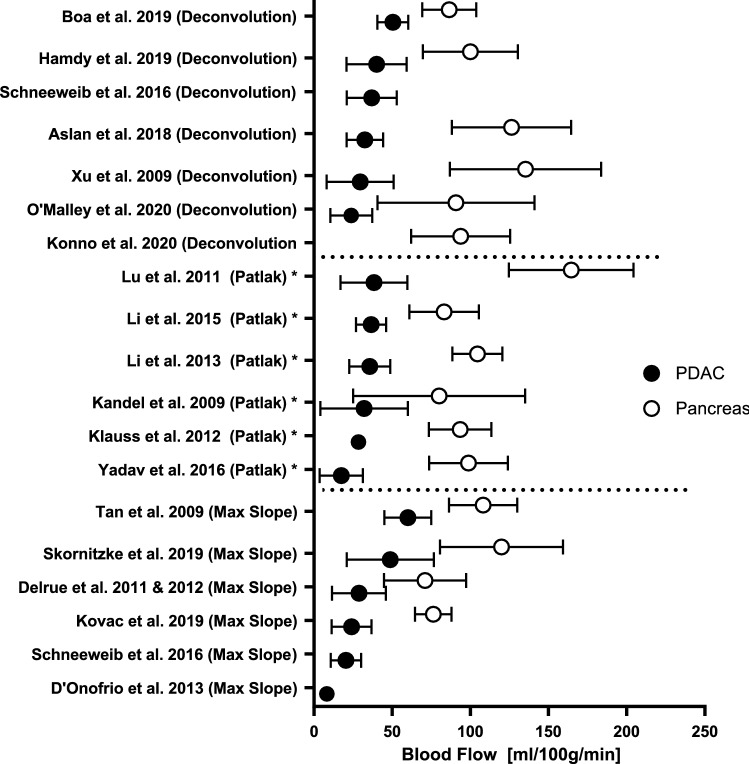
Mean and standard deviation of blood flow of tumor (PDAC) and non-tumorous pancreatic parenchyma in all studies sorted by kinetic model. BF in tumor tissue is lower compared to non-tumorous pancreas parenchyma in all studies. *Patlak model was reported in these studies. However, this model solely is not able to calculate BF

In 15 out of 21 studies, BF was measured in both PDAC and non-tumorous pancreatic parenchyma, including a total of 519 patients. In all these studies, mean blood flow was significantly lower in tumor tissue compared to pancreatic parenchyma outside the tumor or in healthy pancreatic tissue in a control group [22–37]. Mean BF ranged from 17 to 60 mL/100 g/min for PDAC and 71–164 mL/100 g/min for pancreatic parenchyma. All mean BF values can be found in Fig. 2.

**Fig. 3:**
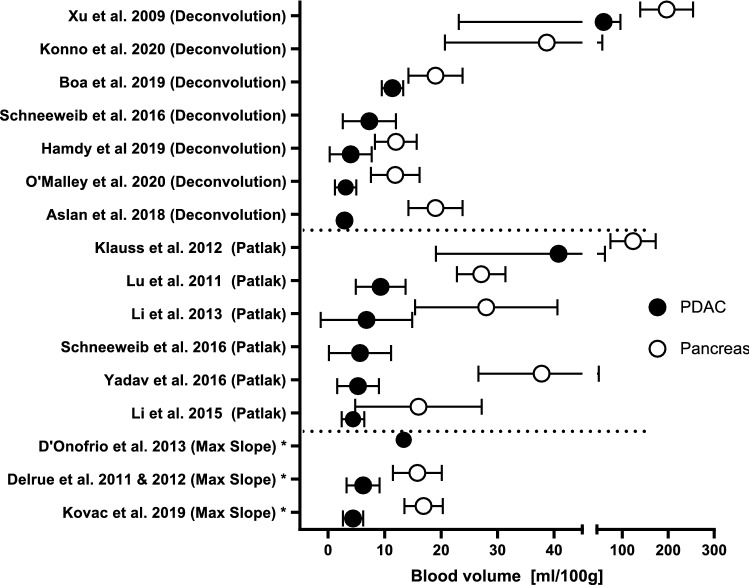
Mean and standard deviation of blood volume of tumor (PDAC) and non-tumorous pancreatic parenchyma in all studies sorted by kinetic model. BV in tumor tissue is lower compared to non-tumorous pancreas parenchyma in all studies. *Maximum slope model was reported in these studies. However, this model solely is not able to calculate BV

In 11 studies, BV was measured for PDAC and non-tumorous pancreatic parenchyma for a total of 423 patients. [22–24, 26, 30, 32, 33, 35–38]. In all of these studies a statistically significantly lower mean BV was found for PDAC compared to pancreatic parenchyma outside the tumor. Mean BV ranged from 2.8 to 59 mL/100 g for PDAC and 15–200 mL/100 g for pancreatic parenchyma. All mean BV values can be found in Fig. 3.

**Fig. 4:**
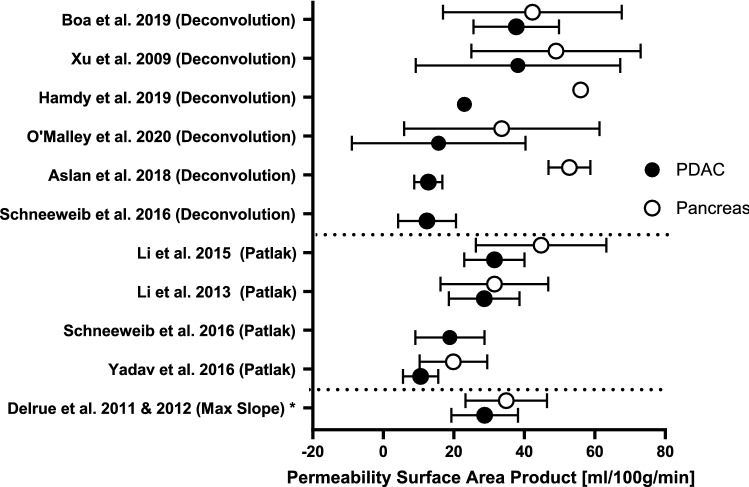
Mean and standard deviation of vascular permeability surface area product (PS) of tumor (PDAC) and non-tumorous pancreatic parenchyma in studies sorted by kinetic model. *Maximum slope model was reported in these studies. However, this model solely is not able to calculate PS

In 9 studies, permeability surface product (PS) was measured for both PDAC and non-tumorous pancreatic parenchyma in a total of 349 patients. In all these studies, the mean permeability was lower in pancreatic tumor tissue [22, 23, 25, 26, 30, 32, 33, 35, 36]. In two studies, this difference was statistically significant [22, 33]. Mean PS for PDAC ranged from 11 to 38 mL/100 g/min and for non-tumorous pancreatic parenchyma from 20 to 56 mL/100 g/min. All mean PS values can be found in Fig. 4.

Aslan et al. and Delrue et al. showed a decrease in perfusion (BF and BV) for both chronic pancreatitis and PDAC. However, compared to acute and chronic pancreatitis, the BF and BV were significantly lower (*p* < 0.01) in PDAC, even for isoattenuating PDAC [22, 37]. Yadav reported that 6/42 lesions isoattenuating on CECT were visible on perfusion color maps. For differentiating PDAC from pancreatitis Yadav et al. report an AUC of 0.83 for BF and 0.80 for BV [33]. Aslan reports both sensitivity and specificity of 100% for BF and BV by using optimal cutoff values [22].

Some studies also assessed semi-quantitative parameters such as peak enhancement and mean transit time (MTT). Peak enhancement showed in two studies to be significantly higher in pancreatic parenchyma than in PDAC (Lu: 30.8 vs 57 HU *p* < 0.016, Tan: 59 vs 101 HU *p* < 0.001) [31, 34]. In two other studies, MTT was significantly higher in PDAC compared to healthy pancreatic parenchyma [Aslan: 11.2 vs. 3.7 s, (*p* < 0.001), Kovac: 7.4 vs. 4 s (*p* < 0.001)] [22, 24].

### Grading

Three articles evaluate the use of CTP to predict histopathological grading of PDAC compared to histopathological diagnosis obtained using a tumor biopsy or resected specimen [24, 39, 40]. These three studies included a total of 124 patients [24, 39, 40].

In two articles, tumors were classified into two groups: low grade (well or moderate differentiated) and high grade (poorly differentiated) [24, 40]. Both studies found higher BV in low-grade tumors compared to high-grade tumors (*p* = 0.001 and *p* = 0.004), whereas a significant difference in BF was only reported by one article (*p* = 0.041). Furthermore, peak enhancement intensity compared to baseline HU was significantly different between high-grade tumors and low-grade tumors, respectively, 16 HU vs. 26 HU. Time to peak (TTP) did not show a significant difference between tumor grades. In addition, a ROC curve analysis was performed to assess the prediction of tumor grade; Kovac et al. calculated an AUC of 0.940 for BF and 0.977 for BV, while D'Onofrio et al. calculated an AUC of 0.798 for BV [24, 40]. Perfusion parameters for the different grades can be found in Table 3.

**Table 3:** For different histopathological grading of PDAC mean/median, BF values are reported in (mL/100 g/min), BV is reported in (mL/100 g), and PS is reported in (mL/100 g/min)

Study	Histopathological grade		
	High	Intermediate	Low grade
Kovac et al. [24]	BF: 17.45 ± 4.1^a^ BV: 2.66 ± 1.0^a^		BF: 28.5 ± 7.7^a^ BV: 5.5 ± 1.4^a^
D'Onofrio et al. [40]	BF: 5.9^b^ BV: 11.3^a, b^		BF: 8.9^b^ BV: 19^a, b^
Schneeweiβ et al. [39] (Max Slope + Patlak)	BF: 21.9 ± 10.4 BV: 5.5 ± 4.5 PS: 11.5 ± 6.4	BF: 21.9 ± 10.4 BV: 5.5 ± 4.5 PS: 21.0 ± 10.2	BF: 20.6 ± 8.6 BV:8.9 ± 11.3 PS:19.3 ± 4.5
Schneeweiβ et al. [39] (Deconvolution)	BF: 35.6 ± 13.9 BV: 6.1 ± 3.9 PS: 11.9 ± 7.1	BF: 37.7 ± 16.6 BV: 7.9 ± 5.5 PS: 13.7 ± 8.8	BF: 33.5 ± 10.3 BV: 6.4 ± 1.3 PS: 11.5 ± 6.4

Kovac and D'Onofrio classified both moderate- and well-differentiated lesions as low grade

^a^Significant differences between pathological grading groups. Grade was high (poorly differentiated), intermediate (moderately differentiated), or low (well differentiated)

^b^Median values, rest of the table report mean values

In the third study, tumors were graded in three groups: well differentiated, moderately differentiated, and poorly differentiated. Using both deconvolution and Patlak combined with Max slope to calculate BF, BV, and PS, no significant differences between the three groups of pathological grade were found [39].

### Treatment response prediction

Two studies investigated the role of CTP as a predictor of treatment response to neoadjuvant chemoradiotherapy with a total of 38 included patients [35, 41]. In both studies, treatment included 45–50,4 Gy radiation in 25–28 fractions and combined gemcitabine-based chemotherapy. Hamdy et al. classified response based on histology of the resection specimen after chemoradiotherapy, whereas Park et al. used the RECIST criteria on conventional CECT after 3 months to assess treatment response; both studies performed CTP before treatment. Hamdy et al. reported a higher pre-treatment BF for responders compared to non-responders (44 vs. 28 mL/100 g/min, *p* = 0.04), whereas PS values were similar [35]. Pre-treatment perfusion measurements of Park et al. showed a significantly higher permeability in responders compared to non-responders (50.8 vs. 19.0 mL/100 mL/min, *p* = 0.01) [41]. In both studies, pre-treatment BV was higher in responders compared to non-responders, although not significant.

Hamdy et al. performed a follow-up CTP after chemoradiation therapy, 7 weeks after baseline CTP. Both BF (*p* = 0.04) and BV (*p* = 0.01) increased significantly in responders after chemoradiotherapy compared to baseline CTP). In non-responders, a non-significant increase in BF (*p* = 0.06) and BV (*p* = 0.06) was reported [35]. Table 4 provides an overview of the perfusion parameters of responders and non-responders.

**Table 4:** Mean/median perfusion parameters of responders and Non-responders during CTP performed at baseline and follow-up after chemoradiotherapy

Study	Baseline responder	Baseline non-responder	FU responder	FU non-responder
Hamdy et al. [35]	BF: (mL/100 g/min): 44^a^ BV: (mL/100 g): 4.3 PS: (mL/100 g/min): 25	BF: 28^a^ BV: 2.0 PS: 20	BF: 54^b^ BV: 6.8^b^ PS: 32	BF: 43 BV: 4.8 PS: 28
Park et al. [41]	Permeability(mL/100 mL/min): 50.8 ± 30.5^a^ BV (mL/100 mL): 5.7 ± 3.0	Permeability: 19.0 ± 10.9^a^ BV: 4.1 ± 1.7		

Parameters of Hamdy reported as median values, parameters of Park reported as mean results

^a^Significant difference between responder and non-responder

^b^Significant difference compared to baseline perfusion parameters of responders. Parameters of Hamdy reported as median values, parameters of Park reported as mean results

Instead of treatment response, another study performed a prediction of survival based on CTP. Perfusion values in peritumoral tissue directly adjacent to tumor tissue were assessed, resulting in a correlation between higher peritumoral blood flow and shorter survival (*p* = 0.004) [42].

### Scanning protocols

In five studies, the main goal was the evaluation of the CTP scan technique for pancreatic cancer. A novel scanning method of an interleaved CTP protocol, where the perfusion acquisition was interleaved with a diagnostic multiphase contrast CECT was introduced in 2013 [43]. This method requires only one contrast bolus, instead of two separate boluses for the perfusion scan and diagnostic scan, allowing for a ‘one-stop-shop’ approach [25, 43, 44]. Two studies demonstrated that simultaneous acquisition of perfusion and high-quality diagnostic images with a single contrast bolus was feasible without difference in quality compared to conventional CECT [25, 44].

Three studies focused on the use of dynamic dual-energy CT acquisitions to calculate perfusion parameters. Li et al. combined both techniques by performing a dual-energy CT after a CTP scan, using the time–attenuation curve to improve the timing of the dual-energy CT [26]. Skornitzke et al. calculated BF based on DECT iodine enhancement images. However, these CTP maps did show a significant improvement compared to conventional acquisitions [29]. Bao et al. showed a good correlation of iodine concentration with both BF and BV, indicating the potential of dual-energy CT to reflect hemodynamic changes using a lower radiation dose [30]. However, the sensitivity to discriminate PDAC from non-tumorous pancreatic parenchyma was higher using CTP parameters with an AUC of 0.971 and 0.958 for BF and BV, compared to AUC of 0.842 for dual-energy-based iodine maps.

### Kinetic models

In the reviewed articles, calculation of perfusion parameters is performed using three main kinetic models: Max slope (one compartment), Patlak (two compartment), and deconvolution.

The maximum slope model assumes a single compartment to estimate the BF using the maximum slope of the time–attenuation curve. In addition, semi-quantitative parameters can be deduced from this time–attenuation curve, like peak enhancement compared to baseline and time to peak [45]. The single-compartment model assumes the absence of venous outflow, therefore perfusion parameters as BV, and PS cannot be estimated [46].

The standard Patlak plot is a linear graphical representation of a two-compartment model, which assumes the distribution of injected contrast agent over two well-mixed compartments. The model is based on an irreversible transfer of contrast agent from the intravascular to the extravascular compartment, allowing estimations of the blood volume and permeability based on the linear part of the Patlak plot [47–49].

In the deconvolution method, the time–attenuation curve of the tissue is assumed to be the convolution between the arterial input function and the impulse residue function. This last curve is a theoretical representation of the fraction of contrast medium that remains in the tissue and can be calculated by deconvolution. On the basis of this impulse residue function, the BF, BV, and MTT are approximated [50–52].

Mean perfusion parameters in PDAC and non-tumorous pancreatic tissue as included in this review show a wide variance as visible in Figs. 2, 3, and 4. When studying the differences between the models, mean BF values calculated with the maximum slope model were significantly lower than using the deconvolution method [20.4 ± 9.7 mL/min/100 g and 36.9 ± 15.6 mL/min/100 g (*p* < 0.004)] [39]. BV values calculated with the deconvolution method resulted in significant higher values than with the Patlak model [7.3 ± 4.7 mL/100 g vs. 5.6 ± 5.5 mL/100 g (*p* < 0.001)], in contrast to the values found for PS [12.4 ± 8.2 mL/100 g/min for deconvolution vs 18.9 ± 9.8 mL/100 g/min for Patlak, (*p* < 0.001)]. However, comparing perfusion parameters of both models, a good correlation was found between Deconvolution and Max slope + Patlak parameters using ICC and Pearson linear correlation coefficient [39].

### Risk of bias

**Fig. 5:**
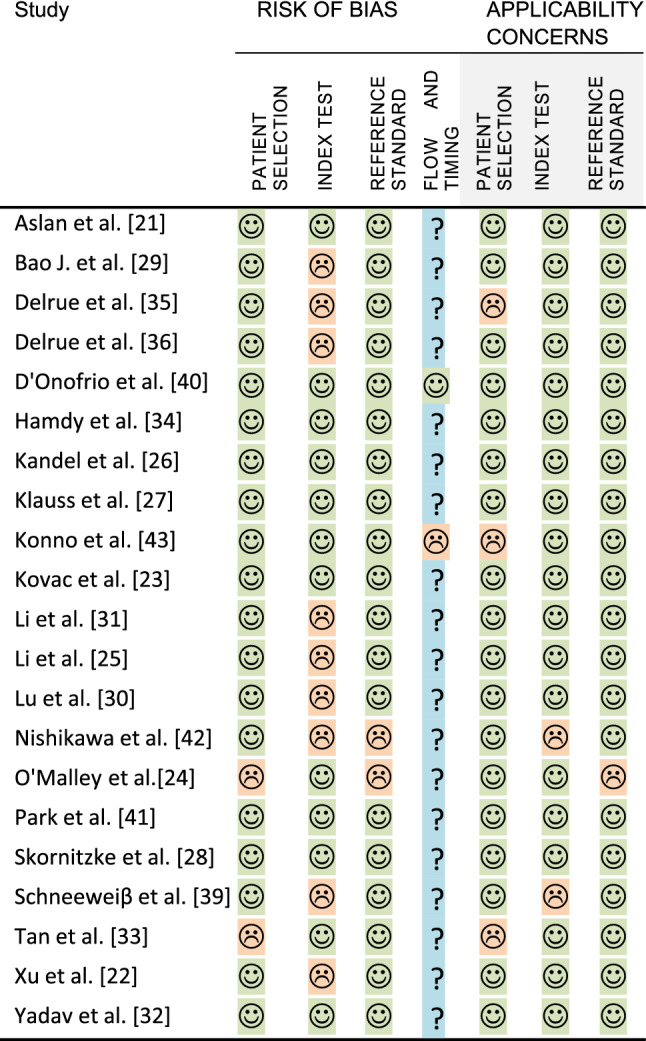
Quality assessment of diagnostic accuracy studies (QUADAS-2)

Figure 5 shows the results of risk of bias and concerns about applicability using the QUADAS-2 tool. Overall studies show a low risk of bias; however, the index test is not always accurately reported, especially concerning the use of the kinetic model. Some studies show an inconsistency, as reported perfusion parameters cannot be calculated from the presented kinetic model, and these studies were marked as high risk in bias for the index test.

Patient flow and timing between CTP and reference standard were reported poorly in almost all studies. For QUADAS-2 per study, see Fig. 4.

## Discussion

### Main study findings

The results from this systematic review show that CTP can accurately distinguish PDAC from non-tumorous pancreatic parenchyma using perfusion parameters calculated with a kinetic model. There is a clear consensus that PDAC can be distinguished from non-tumorous pancreatic parenchyma with a significantly lower BF and BV in PDAC compared to non-tumorous pancreatic parenchyma. Only a few studies showed a significant difference in PS between PDAC and non-tumorous pancreatic parenchyma, but the mean PS for PDAC was consistently lower. Although not studied prospectively, perfusion parameters seem to be able to improve the detection of PDAC that is visually isoattenuating on biphasic CECT.

For grading, CTP can be used as an additional imaging biomarker, which is an important prognostic variable of survival in PDAC. In two studies, a significant difference was found between high-grade tumors and low-grade tumors for BV, and in one of these studies, BF also showed a significant difference [24, 40]. In a third study, no significant difference could be found between three pathological grades using BF, BV, and permeability [39]. There is no clear consensus for the use of CTP as a biomarker for the pathological grade. Nonetheless, two out of three studies show significant differences between grades. Large clinical studies are needed to correlate perfusion parameters to histological grade on the resection specimen.

Treatment assessment using CTP was investigated in two studies. Pre-treatment BF and permeability showed to be a good indicator of histopathological response to chemoradiotherapy [35, 41]. Furthermore, CTP was also performed to assess the effects after chemoradiation therapy. Both responders and non-responders showed an increase in blood flow and blood volume, but only the increase in responders was significant. The microenvironment of PDAC could provide an explanation for these findings, as PDAC has a stroma-rich tumor environment with high intratumoral pressure [52]. This could result in impaired perfusion, eventually impeding the delivery of oxygen and chemotherapy to tumor cells [53]. In tumors with less vessel restriction, reflected by higher baseline perfusion, chemotherapy is better able to penetrate the tissue. Both prediction and assessment of chemoradiotherapy response show promising results which reflect microenvironmental differences. Clinical studies are needed to assess the use of CTP in both baseline and follow-up of treatment, to evaluate CTP as biomarker in treatment assessment.

### Kinetic models/scan parameters

Perfusion parameters strongly depend (up to 30%) on the applied kinetic model and are not directly interchangeable, as shown in a variety of other cancers [51]. Of the included studies, 7 out of 21 used a deconvolution algorithm, 8 used a max-slope algorithm, and in 6 studies, a Patlak analysis was reported. Four studies reported a combination of the compartment models: Max-slope and Patlak analysis. The reported perfusion parameters display a wide range of variability between different kinetic models and perfusion parameters do not always correspond with the reported model. In six articles, BF values were described, even though the reported model was the Patlak model. An explanation could be that some software combines the Patlak and the max-slope models, using the latter to determine the BF. Furthermore, three studies report BV and two PS, although only a max-slope model was mentioned.

In this review, three frequently used kinetic models are described; variations in these models exist, but use is not clearly reported in the literature. The assumptions of the kinetic model influence the calculated quantitative parameters differently. Maximum slope assumes a single compartment and, therefore, no venous outflow out of the tissue. The advantage of this model is the short acquisition time and the simplicity of mathematics. The latter also presents a disadvantage as the correlation between the assumptions made and true physiology is difficult. In all healthy tissue, venous outflow is present; therefore, the true BF is higher than calculated with this model.

The standard Patlak model quantifies the exchange between two compartments: the intravascular and extravascular compartments. This linear model assumes that the back-flux of contrast agent from the extravascular to the intravascular compartment is negligible [49]. This assumption can be applied only during the first pass of contrast agent in tissue and depends on the relative magnitude of blood flow and permeability surface area. However, as PDAC is a hypovascular tumor, the magnitude of blood flow could, therefore, be inadequate to meet this assumption [49, 54]. To take the back-flux into account, a modified Patlak model has been developed, although this non-linear model is more difficult to implement [55, 56].

Deconvolution uses the arterial and time-attenuation curves to calculate the impulse residue function for the tissue. The advantage is that BF, BV, and MTT can be calculated directly with a single model. It is assumed that contrast material is nondiffusible out of the vessels which is not an accurate theory, as there is leakage into the interstitial space [50].

All three models use different assumptions, which do not always accurately reflect pancreatic (tumor) tissue physiology. It is impossible to exactly modulate the kinetics of a contrast agent in tissue, though optimization for specific indications has led to numerous tracer-kinetic models [57, 58]. The preferred model depends on the desired parameters, target area as well as on the acquisition protocol. Since resolution and noise can influence the quantification, sensitivity to noise could explain the lack of consensus on the more mathematically complex parameter PS.

The chosen acquisition and analysis methods strongly influence estimated perfusion parameters. Included studies used a contrast dose in the range of 40–80 mL, with exception of studies using an interleaved protocol in which a weight-based contrast dose was used. The amount of contrast agent volume (50 vs 100 mL) does not substantially change quantitative perfusion parameters for colorectal cancer [59]. The reproducibility of CTP as assessed by Kaufmann et al. showed an interscan variability in the range of 30% [60]. Respiratory motion during scanning is one of the causes for interscan variability, and motion correction methods significantly improve the reproducibility of CTP [61]. Positioning and size of the measured region of interest (ROI) do influence the measured perfusion values. An ROI comprising the entire tumor diameter leads to more stable perfusion measurements, compared to a smaller ROI. As PDAC is known to be heterogeneous, an ROI in a single slice could introduce measurement bias. This is also highlighted in two studies reporting an increase in perfusion values towards the rim of the tumor [25, 36]. A 3D volume of interest would be a more reliable measurement and allows a better understanding of the whole tumor.

### Limitations

For this systematic review, some limitations need to be addressed. First, the number of clinical studies performing CTP in PDAC is still low with too small study populations. The diagnostic accuracy of the technique is not compared to CECT in terms of diagnosis. Second, it is difficult to quantitively compare perfusion parameters of studies using different kinetic models, analysis software, ROI definitions, and scan parameters. As the diversity of the data influences the calculated parameters, no meta-analysis was performed. Third, poor reporting of applied kinetic models could hamper the interpretation of some included studies. Most studies risks of bias were high or unclear, making the summarized evidence limited.

### Future perspectives

CTP provides high spatiotemporal resolution data for quantitative functional information about PDAC which could help in diagnosis, grading, and treatment prediction. The current use of CTP for PDAC is still limited mainly due to concerns regarding technique and knowledge. The amount of radiation for a CTP has been reduced due to improvements in detector efficiency and reconstruction algorithms. The additional information provided by CTP could help steer treatment decision and, therefore, seems to outweigh the risk of radiation.

There are several developments which could help to bring CTP into clinical practice. Advanced interleaved techniques make it possible to perform CTP during routine multiphase contrast-enhanced pancreatic CT using a single-contrast injection [25, 44]. In such a ‘one-stop-shop’ procedure, perfusion information is acquired without extra time and cost for the patient and can be applied in oncologic imaging adding functional information. Furthermore, due to new multidetector CT scanners, larger tissue volumes can be scanned, which facilitate perfusion evaluation of the whole tumors and their surroundings.

One of the barriers limiting widespread adoption seems the lack of reference values, scan standards, and validation. It is difficult to compare perfusion values across different scanners, kinetic models, software, and scan parameters, limiting the use of CTP as a quantitative imaging biomarker for clinical decision making. To increase the use of CTP, some steps need to be taken as described in the EIBALL criteria [62]. First, standardization of scan protocol and reporting of CTP need to be established, enabling better comparison of studies. In addition, perfusion parameters need to be validated with pathology assessment such as microvessel density, permeability, or aggressiveness markers. The latter allows a better understanding of quantitative values aiding in treatment response prediction and, therefore, precision treatment.

Improvement of post-processing also could lead to a more reliable assessment of perfusion parameters. Compartmental and deconvolution analysis are the most widely used kinetic models; however, there is no consensus regarding applicability for abdominal oncology. In this review, we showed that results among studies are not directly comparable. Kinetic models and CTP software are often not tailored and validated for oncology. Because of the physiological differences, other perfusion models and parameters might be more useful to assess tumor characteristics or treatment response.

Novel analysis methods using artificial intelligence (AI) could help extracting diagnostic CTP information from time–intensity curves. AI can facilitate kinetic model-independent interpretation of CTP, reducing inter-observer bias and parameter variability. Radiomics can effectively combine all CTP parameters with spatial information to guide treatment of patients with PDAC [63]. These data-driven biomarkers and their potential to improve tumor characterization and treatment assessment are increasingly investigated [63–65]. A combination of CTP and radiomic features already shows to improve the prediction of response in laryngeal cancer [66]. Furthermore, AI methods and advanced filters could reduce noise in CTP images, improving the image quality and quantitative analysis. For instance, dynamic similarity filters not only are already in use for cardiac CTP but are also in development for abdominal CTP [67]. These developments could facilitate clinical adoption and maximize impact by optimizing the analysis of CTP images regardless of acquisition and reconstruction parameters.

### Conclusion

Quantitative CTP shows a potential benefit in PDAC diagnosis and can serve as a tool for pathological grading and treatment assessment; however, clinical evidence is still limited. To improve clinical use, standardized acquisition and reconstruction parameters are necessary for the interchangeability of the perfusion parameters. The use of an interleaved CTP-CECT protocol followed by post-processing and AI-supported analysis could advance the use of CTP as a predictive biomarker for PDAC.

## Supplementary Information

Below is the link to the electronic supplementary material.

Supplementary file1 (DOCX 19 KB)
