# Supplementary material for: Quantitative CT perfusion imaging in patients with pancreatic cancer: a systematic review
Source: Abdom Radiol (NY). 2021 Jul 5;47(9):3101–17. doi: 10.1007/s00261-021-03190-w (PMC9388409; doi:10.1007/s00261-021-03190-w)
Supplement: Supplementary file 1 — Supplementary file1 (DOCX 19 KB) [file 261_2021_3190_MOESM1_ESM.docx]

**Supplementary Materials:**

Quantitative CT perfusion imaging in patients with pancreatic cancer: A systematic review

T.H. Perik, E.A.J. van Genugten, E.H.J.G. Aarntzen, E.J. Smit, H. Huisman & J.J. Hermans

*Table S1. Search string*

| # | Source | Search string |
| --- | --- | --- |
| 1 | Pubmed | (("Perfusion Imaging"[Mesh] OR Perfusion[tiab] OR CT Perfusion [tiab] OR CTP[tiab] OR DECT [tiab] OR “Dynamic contrast-enhanced” [tiab])) AND ("Tomography, X-Ray Computed"[Mesh] OR imaging[tiab] OR CT[tiab] OR Computer Tomograph*[tiab])  AND  Pancreatic Neoplasms[Mesh] OR Carcinoma, Neuroendocrine[Mesh] OR "Pancreatic neoplasms"[tiab] OR "Pancreatic cancer"[tiab] OR "Pancreatic Adenocarcinoma"[tiab] OR "Pancreatic Ductal adenocarcinoma"[tiab] OR "Neuroendocrine Neoplasms"[tiab] OR "Neuroendocrine Cancer"[tiab] |
| 2 | EMBASE | perfusion imaging.mp. and exp scintigraphy/ or  (CT perfusion or CTP or DECT or dynamic-contrast enhanced).ti,ab,kw.  And exp computer assisted tomography/   \| Or (CT or computed tomograph* or imaging).ti,ab,kw. \| \| --- \|   AND  exp pancreas cancer/ or exp neuroendocrine tumor/ and  (Pancreatic neoplasms or pancreatic cancer or Pancreatic adenocarcinoma or pancreatic ductal adenocarcinoma or neuroendocrine neoplasms or neuroendocrine tumor).ti,ab,kw. |
| 3 | Web of Science | TS = (Perfusion imaging or CT perfusion or CTP or DECT or dynamic-contrast enhanced)  AND TS = (CT or computed tomograph* or “CT-imaging”)  AND  TS = ("Pancreas cancer" or "Pancreatic neoplasm*" or "pancreatic cancer" or"pancreatic adenocarcinoma" or "pancreatic ductal adenocarcinoma" or "neuroendocrine neoplasms" or "neuroendocrine tumor") |

Time range: 01-01-2000 to 31-12-2020
